# Exploration and bioinformatic prediction for profile of mRNA bound to circular RNA BTBD7_hsa_circ_0000563 in coronary artery disease

**DOI:** 10.1186/s12872-024-03711-7

**Published:** 2024-01-24

**Authors:** Ning Guo, Hanxiao Zhou, Qian Zhang, Yahong Fu, Qiaowei Jia, Xiongkang Gan, Yanjun Wang, Shu He, Chengcheng Li, Zhengxian Tao, Jun Liu, Enzhi Jia

**Affiliations:** 1 Department of Cardiovascular Medicine, The First Affiliated Hospital of Nanjing Medical University, Guangzhou Road 300, Nanjing, 210029 Jiangsu Province China; 2 Suzhou Hospital of Integrated Traditional Chinese and Western Medicine, Suzhou, 215101 Jiangsu Province China; 3 Department of Cardiology, Jurong City People’s Hospital, Ersheng Road 66, Jurong, 212400 Jiangsu Province China

**Keywords:** Coronary artery disease, PBMC, BTBD7_hsa_circ_0000563, ChIRP-RNAseq

## Abstract

**Background:**

As a novel circRNA, BTBD7_hsa_circ_0000563 has not been fully investigated in coronary artery disease (CAD). Our aim is to reveal the possible functional role and regulatory pathway of BTBD7_hsa_circ_0000563 in CAD via exploring genes combined with BTBD7_hsa_circ_0000563.

**Methods:**

A total of 45 peripheral blood mononuclear cell (PBMC) samples of CAD patients were enrolled. The ChIRP-RNAseq assay was performed to directly explore genes bound to BTBD7_hsa_circ_0000563. The Gene Ontology (GO) and Kyoto Encyclopedia of Genes and Genomes (KEGG) analysis were conducted to reveal possible functions of these genes. The interaction network was constructed by the STRING database and the Cytoscape software. The Cytoscape software were used again to identify clusters and hub genes of genes bound to BTBD7_hsa_circ_0000563. The target miRNAs of hub genes were predicted via online databases.

**Results:**

In this study, a total of 221 mRNAs directly bound to BTBD7_hsa_circ_0000563 were identified in PBMCs of CAD patients via ChIRP-RNAseq. The functional enrichment analysis revealed that these mRNAs may participate in translation and necroptosis. Moreover, the interaction network showed that there may be a close relationship between these mRNAs. Eight clusters can be further subdivided from the interaction network. RPS3 and RPSA were identified as hub genes and hsa-miR-493-5p was predicted to be the target miRNA of RPS3.

**Conclusions:**

BTBD7_hsa_circ_0000563 and mRNAs directly bound to it may influence the initiation and progression of CAD, among which RPS3 and RPSA may be hub genes. These findings may provide innovative ideas for further research on CAD.

**Supplementary Information:**

The online version contains supplementary material available at 10.1186/s12872-024-03711-7.

## Background

Coronary artery disease (CAD) still poses a great threat to human health and quality of lives, although the current treatment strategies for CAD are quite mature [1, 2]. Since CAD is considered to be influenced by a combination of genetic and lifestyle factors, deeper genetic factors deserve to be explored [3].

In eukaryotes, circular RNAs (circRNAs) are covalently closed and endogenous biomolecules without 5′ and 3′ ends and poly-a-tail. The synthesis of circRNAs relies on reverse splicing, which includes connection between the donor site and the upstream receptor site, making circRNAs containing a single exon or multiple exons [4]. Currently, the functions of circRNAs that have been revealed mainly include “microRNAs (miRNAs) sponges”, regulation of transcription, combination with RNA binding proteins (RBPs), and encoding small peptides [4–6]. Moreover, circRNAs have been shown to not only serve as biomarkers to assist diagnosis in diseases such as cerebrovascular disease, various cancers, and liver disease [7–9], but also as therapeutic targets for these diseases [9–11]. Due to the characteristics of expression abundant and evolutionarily conserved, the impact of circRNAs on the pathogenesis of CAD is gradually being emphasized and research on the mechanism of circRNAs involvement in CAD is rapidly developing as well [12, 13]. However, it remains unclear whether circRNAs can interact with RNAs other than miRNAs to regulate gene expression.

In our previous study, we found that the expression level of BTBD7_hsa_circ_0000563 was significantly lower in human coronary artery samples with severe atherosclerotic stenosis than that with moderate atherosclerotic stenosis [14]. Simultaneously, we observed that the level of BTBD7_hsa_circ_0000563 was significantly lower in PBMCs of CAD patients compared to healthy controls [15]. However, it is crucial to investigate the function and regulatory pathway of BTBD7_hsa_circ_0000563 to explore the relationship between BTBD7_hsa_circ_0000563 and CAD.

In the present investigation, we profiled the expression of transcriptome-wide RNAs bound to BTBD7_hsa_circ_0000563 (except for miRNA) in peripheral blood mononuclear cells (PBMCs) of subjects with CAD by means of chromatin isolation by RNA purification followed by RNA sequencing (ChIRP-RNAseq). Moreover, bioinformatics analyses were conducted to predict functions of RNAs bound to BTBD7_hsa_circ_0000563.

## Materials and methods

### Study subjects

From May 2021 to October 2021, a total of 45 subjects with CAD were enrolled from the First Affiliated Hospital of Nanjing Medical University. Subjects with infectious diseases, malignant neoplasms, cerebrovascular disease, congenital heart disease, myocardiopathy, rheumatic valvular disease, and dysfunction with adrenal, liver, kidney or thyroid were excluded from the present study. All subjects underwent coronary artery angiography. According to the ACC/AHA classification, the diagnosis criteria of CAD as following: stenosis of major epicardial coronary artery ≥50% [16]. The baseline characteristics of the subjects were shown in Table 1.

**Table 1:** Baseline characteristics of the study subjects

Characteristic	CAD (*n* = 45)
Age (years)	61.98 ± 9.82
Sex (male/female)	29/16
BMI (kg/m2)	25.35 ± 3.24
SBP (mmHg)	131.89 ± 20.66
DBP (mmHg)	80.67 ± 12.10
Hypertension (n, %)	33 (73.33)
Smoking (n, %)	25 (55.56)
Drinking (n, %)	15 (33.33)
TC (mmol/L)	3.95 (3.06–4.73)
TG (mmol/L)	1.58 ± 0.60
HDL-C (mmol/L)	1.03 ± 0.22
LDL-C (mmol/L)	2.52 (1.76–3.08)
Fasting blood glucose (mmol/L)	5.09 (4.69–5.70)
Serum creatinine (umol/L)	72.70 ± 15.97
Gensini score	40.50 (19.50–78.50)
Coronary artery disease	
1 vessel (n, %)	14 (31.11)
2 vessels (n, %)	12 (26.67)
3 vessels (n, %)	17 (37.78)
Other vessels (n, %)	2 (4.44)

1vessel, stenosis of any single vessel in LAD, LCX, and RCA ≥50%; 2 vessels, stenosis of any two vessels in LAD, LCX, and RCA ≥50%; 3 vessels, stenosis of LAD, LCX, and RCA or stenosis of LM and RCA ≥50%; other vessels, diagonal branch and obtuse marginal branch

*CAD* coronary artery disease, *BMI* body mass index, *SBP* systolic blood pressure, *DBP* diastolic blood pressure, *TC* total cholesterol, *TG* triacylglycerol, *HDL-C* high-density lipoprotein cholesterol, *LDL-C* low-density lipoprotein cholesterol, *LAD* left anterior descending branch, *LCX* left circumflex branch, *RCA* right coronary artery, *LM* left main coronary artery

Written informed consent from all subjects was obtained. All experimental protocols were approved by the ethics committee of the First Affiliated Hospital of Nanjing Medical University and were conducted in accordance with the Declaration of Helsinki.

### PBMCs isolation

Ten ml of artery blood samples were drawn from the arterial sheaths in all subjects by artery puncture before coronary angiography. After being treated with Lymphocyte Separation Medium (TBD, Tianjin, China), PBMCs were isolated from the diluted whole blood to the middle white monolayer by density gradient centrifugation. Next, after repeated centrifugations, PBMCs were completely separated and precipitated. The detailed process is provided in Additional file 1. The isolated PBMC samples were preserved at − 80 °C until use.

### Chromatin isolation by RNA purification (ChIRP) assays

The ChIRP assay was used to study the interaction between BTBD7_hsa_circ_0000563 and RNA on a genome-wide scale [17]. Four probes targeting the splice-junction sequence of BTBD7_hsa_circ_0000563 were designed and synthesized by Ribobio Co., Ltd. (Ribobio, China) (Probe_1 CATTTCTTGTGCAAGCATGT−/3bio/; Probe_2 TCATCCATTTCTTGTGCAAG−/3bio/; Probe_3 CCATTTCTTGTGCAAGCATG−/3bio/; Probe_4 ATCCATTTCTTGTGCAAGCA−/3bio/). The specificity and accuracy of the BTBD7_hsa_circ_0000563 probes were verified in our previous study [15].

Forty-five PBMC samples were mixed into one. RNA bound to BTBD7_hsa_circ_0000563 was harvested using ChIRP kit (Gzscbio, China) according to instructions of the manufacturer. First, after crosslinking with 1% formaldehyde at room temperature for 10 min, PBMCs were lysed using lysis buffer. Second, a total of 5 μl lysates were taken from the cleaned lysates as the input group for standby, and 500 μl lysates were taken from the cleaned lysates as the target group which were hybridized with 100 pmol biotinylated BTBD7_hsa_circ_0000563 specific probes at 37 °C for 4 h. Third, after 4-hour hybridization, streptavidin magnetic beads were hybridized with the mixture of lysates and probes at 37 °C for 30 min. Finally, the input group and eluted and purified products of the target group were applied to isolation of RNA. And the isolated RNA was used to construct the RNA sequencing library.

### Library preparation and high-throughput sequencing

RNA quality control was conducted using NanoDrop 2000 (Thermo Fisher Scientific, USA). RNA libraries were constructed using NEBNext® Ultra™ RNA Library Prep Kit for Illumina® (NEB, USA) according to the manufacturer’s instructions and were purified using AMPure beads (Beckman Coulter, USA). The quality and quantity of libraries were evaluated using Ailgent 4200 (Ailgent Technologies, USA). Sequencing was performed using illumina Novaseq 6000 (Illumina, USA) with 150 bp paired end reads. Then, raw data was obtained from sequencing. After removing adapter-containing and low-quality reads from the raw data, clean data was obtained. Quality control of the raw data and clean data were completed by FastQC (http://www.bioinformatics.babraham.ac.uk/projects/fastqc/), and the summarized plots of quality control reports were created by MultiQC (v 1.11) [18].

### Identification of differentially expressed genes

Clean reads were mapped to the human reference genome (UCSC hg38) via HISAT2 software (v 2.2.0) [19]. In order to quantify high-quality mapped reads, featureCounts tool of subread software (v 2.0.1) [20] was employed. Then, edgeR software (v 3.16) was used to normalize the original readcount and calculate the fold change, *P*-value, and false discovery rate (FDR) of RNAs between the target group and the negative control group. RNAs that met the criterion of |log2(FoldChange)| > 1 and FDR < 0.05 were defined as differentially expressed genes (DEGs).

### Functional enrichment analysis

Gene Ontology (GO) functional enrichment and Kyoto Encyclopedia of Genes and Genomes (KEGG) pathway analyses of upregulated DEGs and genes of clusters were performed using the clusterProfiler 4.0 package in R [21]. The ggplot2 package and the DOSE package [22] were employed to visualize enrichment results in R.

### Interaction network and clusters construction and hub genes analysis

The protein–protein interaction (PPI) analysis of upregulated DEGs was performed using the STRING database (STRING v11.5) (https://string-db.org/). Simultaneously, the interaction data obtained from STRING was loaded into the Cytoscape software (v 3.9.1) to structure interaction networks. In the Cytoscape software, the cytoHubba tool [23] was used to calculate the candidate hub genes and the cytoCluster tool [24] was used to make cluster analysis. Thereafter, candidate target miRNAs of hub genes were predicted via the TargetScan database [25] (TargetScanHuman v8.0) (https://www.targetscan.org/vert_80/), the miRTargBase database [26] (miRTargBase v9.0) (https://mirtarbase.cuhk.edu.cn/~miRTarBase/miRTarBase_2022/php/index.php), and the StarBase database [27] (https://starbase.sysu.edu.cn/). In addition, Venn diagrams were generated by EVenn online (http://www.ehbio.com/test/venn/#/) [28] to figure out intersection of different gene sets.

### Statistical analysis

Categorical variables are displayed as counts (percentages). Normally distributed continuous variables are presented as mean ± standard deviation, while non-normally distributed continuous variables are described as median (25th–75th interquartile range). All data analyses were performed using SPSS 21 software. Two-tailed *P* values < 0.05 were considered to be statistically significant.

## Results

### Quality control of the sequencing data

**Fig. 1:**
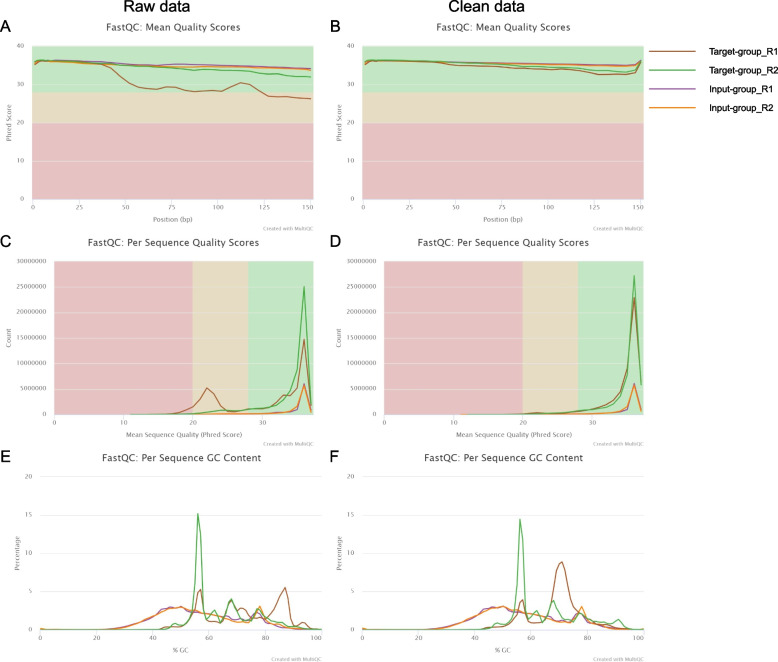
The quality control of the sequencing data. **A** Mean quality score of each group in raw data. The Green area represents excellent quality. The yellow area represents medium quality. The red area represents poor quality. **B** Mean quality score of each group in clean data. **C** Per sequence quality score of each group in raw data. **D** Per sequence quality score of each group in clean data. **E** Per sequence GC content of each group in raw data. **F** Per sequence GC content of each group in clean data

Through ChIRP-RNAseq assay, a total of 55,004,477 raw reads and 10,292,036 raw reads were obtain from the target group and the input group, respectively. After removing adapter-containing and low-quality reads, a total of 53,528,122 clean reads in the target group and 10,003,634 clean reads in the input group were left. The quality control results of raw data and clean data were visualized and presented in Fig. 1. The mean phred scores of each position of reads were almost higher than 30 in all groups (Fig. 1A and B). Moreover, Q30 percentage of input group was > 90% while Q30 percentage of the target group was < 90% (read1 = 61.94%, read2 = 87.16%) in raw data. Nevertheless, in clean data, Q30 percentages of all groups were > 93% (Fig. 1C and D), which indicated that the quality of clean data was high. Additionally, the average GC content of each sample was between 55 and 70% (Fig. 1E and F). Furthermore, properly mapped ratios of the target group and the input group reached 81.71 and 87.63%, respectively. To sum up, our results of ChIRP-RNAseq assay and relevant analysis were reliable.

### Identification of mRNA bound to BTBD7_hsa_circ_0000563

**Fig. 2:**
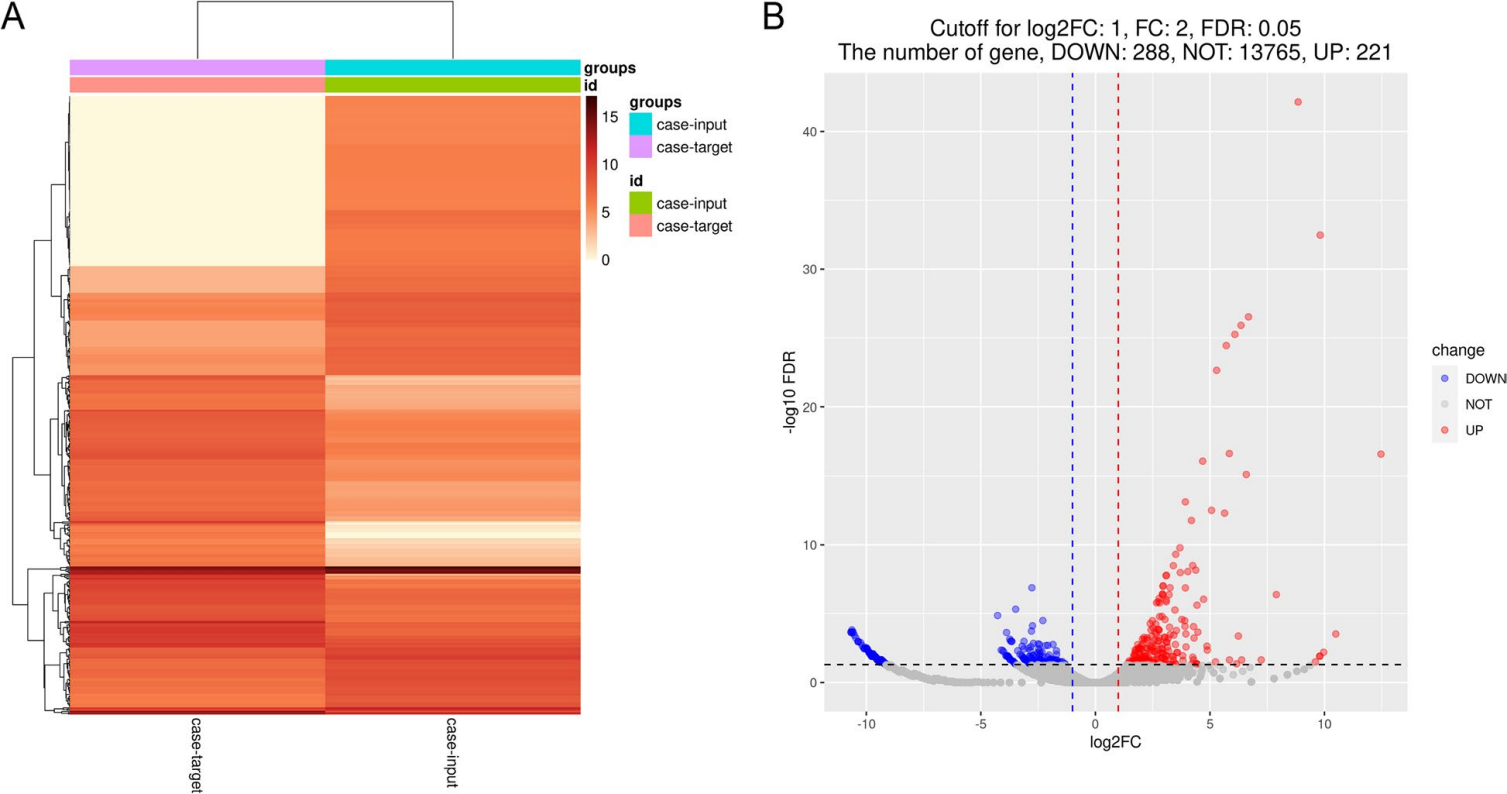
The differentially expressed genes. **A** The heatmap of differentially expressed genes without row scale. **B** The volcano plot of differentially expressed genes (Cutoff: FC = 2, log_2_FC = 1, FDR = 0.05). FC, fold change; FDR, false discovery rate

After normalizing and comparing expression levels of mRNAs captured by probes between the target group and the input group, a total of 509 differentially expressed mRNAs were found, among which 221 mRNAs were upregulated and 288 mRNAs were downregulated in the target group (Fig. 2). Compared with the input group, mRNAs upregulated in the target group were identified as mRNAs directly combined with BTBD7_hsa_circ_0000563 in PBMC of CAD patients through the ChIRP-RNAseq assay, because the input group, as the control group, was used to eliminate the background factors and the unavoidable systematical errors. Therefore, subsequent analyses would be based on mRNAs upregulated in the target group. In addition, to be intuitionistic, top 30 upregulated mRNAs were presented in Table 2.

**Table 2:** Top 30 mRNAs bound to BTBD7_hsa_circ_0000563

Gene symbol	Ensembl	FDR	Log_2_FC	Biotype	Gene location
BARD1	ENSG00000138376	7.21 × 10^−43^	8.85	protein_coding	chr2:214725646–214,809,711
IL6	ENSG00000136244	3.34 × 10^−33^	9.81	protein_coding	chr7:22725884–22,732,002
METTL12	ENSG00000214756	2.94 × 10^−27^	6.68	protein_coding	chr11:62665309–62,668,496
TTI2	ENSG00000129696	1.22 × 10^−26^	6.36	protein_coding	chr8:33473386–33,513,601
TAF1D	ENSG00000166012	5.55 × 10^−26^	6.09	protein_coding	chr11:93729948–93,784,391
EIF4A1	ENSG00000161960	3.57 × 10^−25^	5.71	protein_coding	chr17:7572706–7,579,005
WDR74	ENSG00000133316	2.25 × 10^−23^	5.29	protein_coding	chr11:62832342–62,841,809
TMEM107	ENSG00000179029	2.32 × 10^−17^	5.85	protein_coding	chr17:8173237–8,176,399
POU3F3	ENSG00000198914	2.56 × 10^−17^	12.47	protein_coding	chr2:104855511–104,858,574
USB1	ENSG00000103005	8.30 × 10^−17^	4.68	protein_coding	chr16:57999546–58,021,618
HIST2H2AB	ENSG00000184270	7.71 × 10^−16^	6.59	protein_coding	chr1:149887524–149,887,916
RPL13A	ENSG00000142541	7.52 × 10^−14^	3.93	protein_coding	chr19:49487554–49,492,308
HIST2H2AC	ENSG00000184260	3.10 × 10^−13^	5.07	protein_coding	chr1:149886975–149,887,364
PTCH2	ENSG00000117425	4.96 × 10^−13^	5.64	protein_coding	chr1:44819844–44,843,063
ZFHX3	ENSG00000140836	1.69 × 10^−12^	4.19	protein_coding	chr16:72782885–73,144,447
RPL27A	ENSG00000166441	1.63 × 10^−10^	3.69	protein_coding	chr11:8682411–8,714,759
RPSA	ENSG00000168028	4.92 × 10^−10^	3.51	protein_coding	chr3:39406689–39,412,542
H1FX	ENSG00000184897	3.22 × 10^−09^	4.25	protein_coding	chr3:129314771–129,316,277
LYZ	ENSG00000090382	3.26 × 10^−09^	3.40	protein_coding	chr12:69348341–69,354,234
FRAT2	ENSG00000181274	6.84 × 10^−09^	4.38	protein_coding	chr10:97332497–97,334,709
OTUD1	ENSG00000165312	8.75 × 10^−09^	4.03	protein_coding	chr10:23439458–23,442,390
RPLP2	ENSG00000177600	1.04 × 10^−08^	3.71	protein_coding	chr11:809647–812,880
EIF4A2	ENSG00000156976	1.61 × 10^−08^	3.10	protein_coding	chr3:186783205–186,789,900
RACK1	ENSG00000204628	1.76 × 10^−08^	3.09	protein_coding	chr5:181236909–181,248,096
RPL13	ENSG00000167526	9.37 × 10^−08^	2.96	protein_coding	chr16:89560657–89,566,828
KLF2	ENSG00000127528	1.03 × 10^−07^	2.95	protein_coding	chr19:16324817–16,327,874
PPP3R1	ENSG00000221823	1.30 × 10^−07^	3.26	protein_coding	chr2:68178857–68,256,237
APOL1	ENSG00000100342	1.35 × 10^−07^	3.93	protein_coding	chr22:36253010–36,267,530
ARL4C	ENSG00000188042	3.82 × 10^−07^	2.93	protein_coding	chr2:234493041–234,497,053
RPS3	ENSG00000149273	3.82 × 10^−07^	2.93	protein_coding	chr11:75399486–75,422,280

*FDR* false discovery rate, *FC* fold change, *chr* chromosome

### GO enrichment analysis for mRNA bound to BTBD7_hsa_circ_0000563

**Fig. 3:**
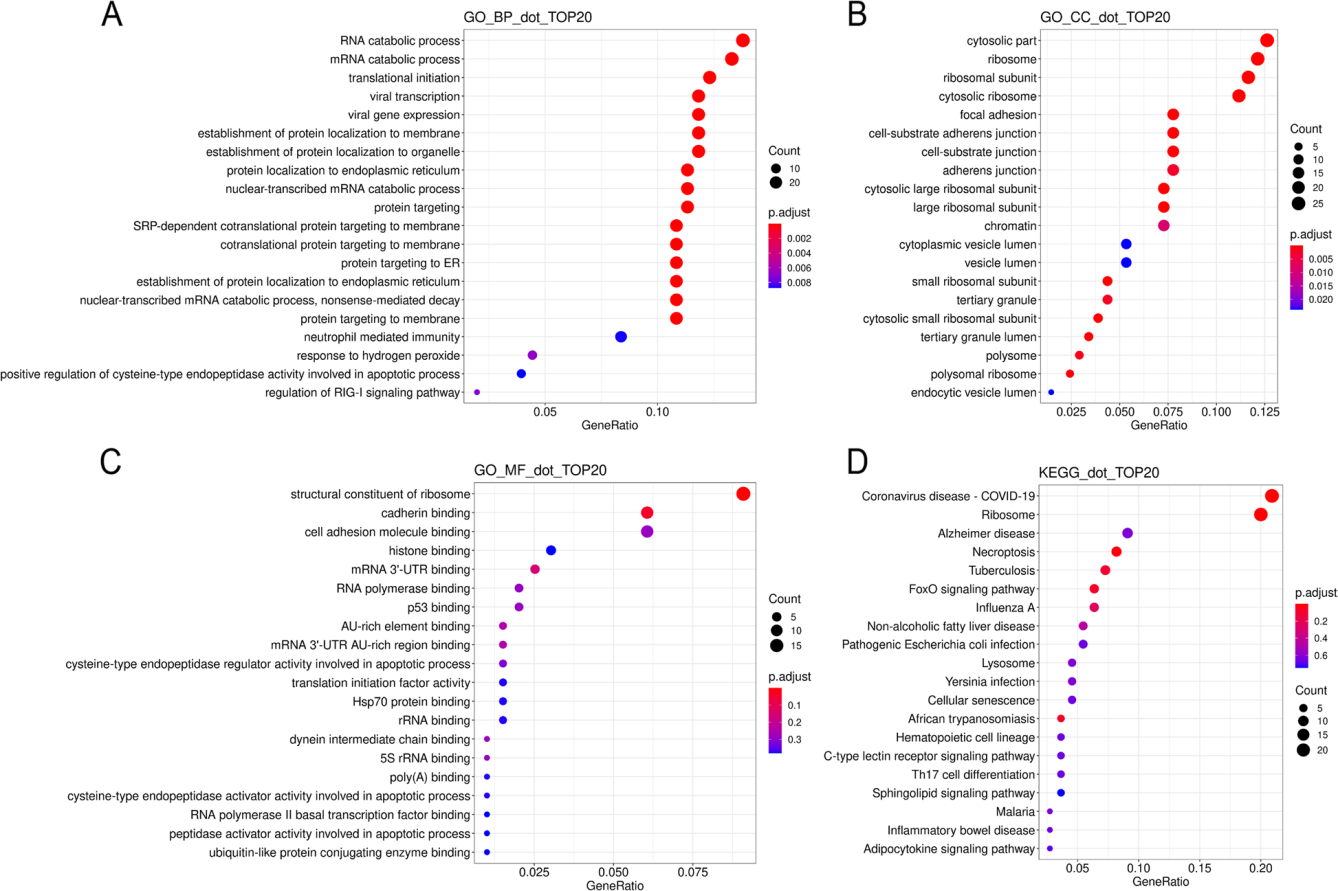
The functional enrichment analysis for mRNAs bound to BTBD7_hsa_circ_0000563. **A** The top 20 biological process terms of GO analysis for mRNAs bound to BTBD7_hsa_circ_0000563. **B** The top 20 cellular component terms of GO analysis for mRNAs bound to BTBD7_hsa_circ_0000563. **C** The top 20 molecular function terms of GO analysis for mRNAs bound to BTBD7_hsa_circ_0000563. **D** The top 20 pathways of KEGG analysis for mRNAs bound to BTBD7_hsa_circ_0000563. GO, Gene Ontology; KEGG, Kyoto Encyclopedia of Genes and Genomes

The GO enrichment analysis was used to reveal possible biological roles of those mRNAs directly bound to BTBD7_hsa_circ_0000563 from three aspects: biological process (BP), cellular component (CC), and molecular function (MF). Top 20 terms of BP, CC, and MF were shown in Fig. 3. Especially, the terms with top 3 adjusted *P* value of each part and their details were listed as follows: BP (Fig. 3A): SRP-dependent cotranslational protein targeting to membrane (GO:0006614, p.adjust = 9.69 × 10^−19^), cotranslational protein targeting to membrane (GO:0006613, p.adjust = 9.69 × 10^−19^), protein targeting to ER (GO:0045047, p.adjust = 1.00 × 10^−18^); CC (Fig. 3B): cytosolic ribosome (GO:0022626, p.adjust = 3.02 × 10^−22^), cytosolic part (GO:0044445, p.adjust = 6.17 × 10^−18^), ribosomal subunit (GO:0044391, p.adjust = 1.15 × 10^−17^); MF (Fig. 3C): structural constituent of ribosome (GO:0003735, p.adjust = 1.40 × 10^−10^), cadherin binding (GO:0045296, p.adjust = 0.041), mRNA 3′-UTR binding (GO: 0003730, p.adjust = 0.152).

### KEGG enrichment analysis for mRNA bound to BTBD7_hsa_circ_0000563

In order to further evaluate the biological significance of these mRNAs directly bound to BTBD7_hsa_circ_0000563, the KEGG enrichment analysis was conducted (Fig. 3D). According to the adjusted *P* value, the top five pathways were ribosome (hsa03010, p.adjust = 1.84 × 10^−14^), coronavirus disease - COVID-19 (hsa05171, p.adjust = 3.33 × 10^−12^), necroptosis (hsa04217, p.adjust = 0.019), African trypanosomiasis (hsa05143, p.adjust = 0.073), and FoxO signaling pathway (hsa04068, p.adjust = 0.076).

### Interaction network construction and cluster analysis for mRNA bound to BTBD7_hsa_circ_0000563

**Fig. 4:**
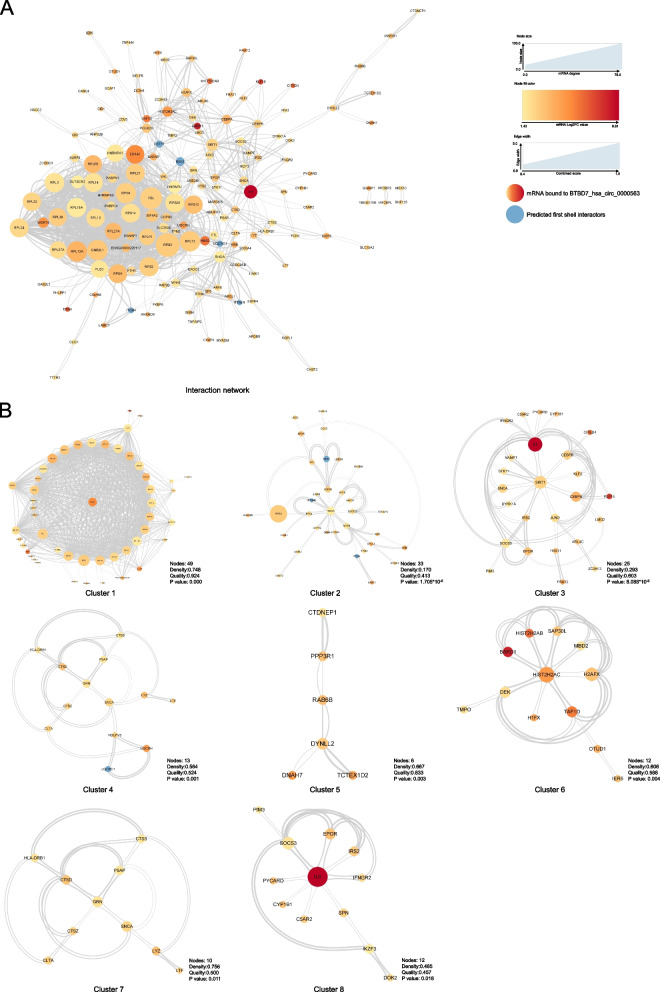
The interaction network and clusters of mRNAs bound to BTBD7_hsa_circ_0000563. **A** The interaction network of mRNAs bound to BTBD7_hsa_circ_0000563. Each node indicates one gene and each edge indicates one interaction between genes. The larger the size of the node is, the greater the degree value of the gene in the interaction network is. The redder the node color is, the greater the log_2_FC value of the gene is. The thicker the edge is, the more reliable the data support of the relationship between genes is. **B** Eight clusters with a *P* value < 0.05 extracted by the cytoCluster tool. FC, fold change

The String database was used to calculate the interaction degree between mRNAs bound to BTBD7_hsa_circ_0000563. Before calculating, the minimum required interaction score was set to 0.40 and the max number of first shell interactors to show was set to no more than 5 interactors. As a result, a mRNA interaction network composed of 158 nodes and 660 edges was constructed using the Cytoscape software (Fig. 4A). The *P* value of the interaction network was < 1.00 × 10^−16^. From this network, we can find out that there was one large cluster with close internal relations, among which RPS3 has the highest node degree.

In order to further calculate main clusters of this interaction network, the cytoCluster tool in the Cytoscape software was used in following analyses. Through merging highly overlapping mRNA nodes (the overlap threshold set to default value 0.8), the ClusterONE algorithm of the cytoCluster tool output a total of 13 clusters that contained no less than three mRNAs and whose density was larger than 0.05, among which 8 clusters had a *P* value < 0.05 (Fig. 4B). The nodes of these clusters were attributed using yFiles radial layout algorithm, which showed that the 8 clusters might respectively centered on EIF4A1 (Cluster 1), RHOA (Cluster 2), SIRT1 (Cluster 3), GRN (Cluster 4 and 7), DYNLL2 (Cluster 5), HIST2H2AC (Cluster 6), IL6 (Cluster 8).

### Functional enrichment analysis for clusters

**Fig. 5:**
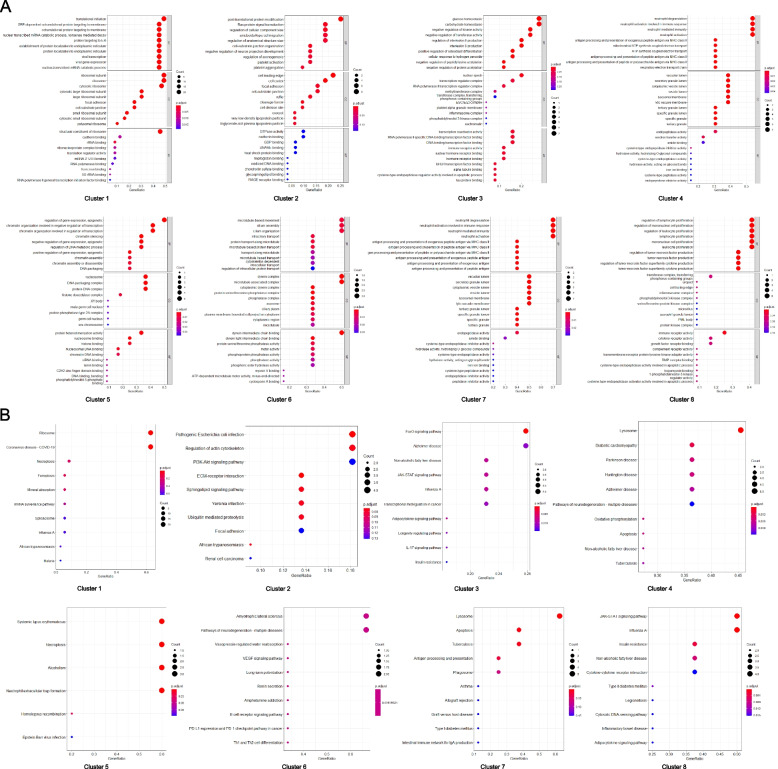
The functional enrichment analysis for clusters. **A** Results of GO analysis for clusters. **B** Results of KEGG analysis for clusters. GO, Gene Ontology; KEGG, Kyoto Encyclopedia of Genes and Genomes

Considering that these clusters separated from the whole interaction network can refine the relationship between genes, GO and KEGG functional enrichment analyses were performed again for the 8 clusters. The results of GO analyses revealed that the 8 clusters were most likely to participate in translational initiation (Cluster 1), post-translational protein modification (Cluster 2), glucose homeostasis (Cluster 3), neutrophil degranulation (Cluster 4 and 7), regulation of gene expression, epigenetic (Cluster 5), microtubule-based movement (Cluster 6), regulation of lymphocyte proliferation (Cluster 8), and other biological processes, respectively (Fig. 5A). Furthermore, the outcomes of KEGG analyses indicated that the 8 clusters were most likely to take part in ribosome (Cluster 1), Pathogenic *Escherichia coli* infection (Cluster 2), FoxO signaling pathway (Cluster 3), lysosome (Cluster 4 and 7), necroptosis (Cluster 5), Amyotrophic lateral sclerosis (Cluster 6), JAK-STAT signaling pathway (Cluster 8), and other pathways, respectively (Fig. 5B).

### Identification of hub genes and target miRNA prediction for hub genes

**Fig. 6:**
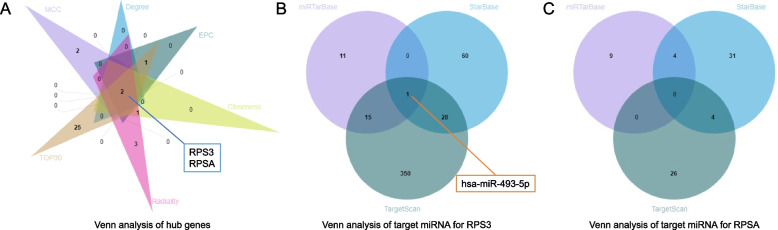
Venn diagrams for screening hub genes and target miRNA. **A** The Venn diagram of five top 10 candidate hub genes sets respectively calculated by the MCC, Degree, EPC, Closeness, and Radiality algorithms and top 30 mRNA bound to BTBD7_hsa_circ_0000563. The intersection contains RPS3 and RPSA. **B** The Venn diagram of three candidate target miRNA sets of RPS3 respectively obtained from the TargertScan, miRTargBase, and StarBase databases. The intersection contains hsa-miR-493-5p. **C** The Venn diagram of three candidate target miRNA sets of RPSA respectively obtained from the TargertScan, miRTargBase, and StarBase databases. There is no intersection between these candidate sets. MCC, Maximal Clique Centrality; EPC, Edge Percolated Component; RPS3, ribosomal protein S3; RPSA, ribosomal protein SA

According to the global-based method, five algorithms were performed respectively to calculate the top 10 genes, namely the Maximal Clique Centrality (MCC) algorithm, the Degree algorithm, the Edge Percolated Component (EPC) algorithm, the Closeness algorithm, and the Radiality algorithm of the cytoHubba tool. After intersecting the above predicted genes and the top 30 genes bound to BTBD7_hsa_circ_0000563, RPS3 and RPSA were identified as hub genes (Fig. 6A). The detailed information of the two hub genes were presented in Table 3.

**Table 3:** The detailed information of the two hub genes

Symbol	ENSEMBL	Description	FDR	Log_2_FC
RPS3	ENSG00000149273	ribosomal protein S3	3.82 × 10^−07^	2.93
RPSA	ENSG00000168028	ribosomal protein SA	4.92 × 10^−10^	3.51

*FDR* false discovery rate, *FC* fold change

In order to obtain highly reliable target miRNA of hub genes, intersections of results from the TargertScan database, the miRTargBase database, and the StarBase database were identified as target miRNA of hub genes. Ultimately, hsa-miR-493-5p was identified as target miRNA of RPS3 (Fig. 6B), while there was no predicted target miRNA of RPSA that met the above requirements (Fig. 6C).

## Discussion

In this investigation, through ChIRP-RNAseq assay, we revealed the profile of RNA bound to BTBD7_hsa_circ_0000563 in PBMCs of CAD patients. A total of 221 mRNAs were identified to bind directly to BTBD7_hsa_circ_0000563. GO and KEGG functional enrichment analyses suggested that these mRNAs may influence CAD via involving in translation and necroptosis processes. We further constructed an interaction network of mRNAs directly bound to BTBD7_hsa_circ_0000563, which showed that there may be a close relationship between these mRNAs. Through more in-depth analyses of this interaction network, we separated eight clusters with a *P* value < 0.05 and calculated that RPS3 and RPSA may be hub genes. Functional enrichment analyses of eight clusters indicated that genes of these clusters may participate in translation, regulation of gene expression, regulation of immune cells, and other processes. After synthesizing the results of multiple databases, hsa-miR-493-5p was identified as target miRNA of RPS3, while RPSA had none.

BTBD7_hsa_circ_0000563 was first reported in the circRNAs profile of animals by Memczak et al. [29]. Subsequently, scientists also discovered BTBD7_hsa_circ_0000563 in normal human tissues [30–32]. BTBD7_hsa_circ_0000563, a circRNA with 1268 bases, is located on chromosome 14. The gene code of BTBD7_hsa_circ_0000563 starts at position 93,760,203 and ends at position 93,762,503 according to the Circbank database (http://www.circbank.cn/). Available studies have corroborated that BTBD7_hsa_circ_0000563 can inhibit adipogenesis [33] and inactivate the p38 mitogen-activated protein kinase (MAPK) signaling pathway [34] by acting as competitive endogenous RNAs (ceRNAs). Moreover, based on the Circbank database, BTBD7_hsa_circ_0000563 has Open Reading Frame (ORF) and internal ribosome entry site (IRES), indicating its enormous potential for encoding polypeptides. The diagnostic value and RBPs of BTBD7_hsa_circ_0000563 in CAD were verified in our previous studies [14, 15]. Recently, multiple studies in various areas introduce ChIRP-seq technology to explore genes directly bound to noncoding RNA (ncRNA) [17, 35–37]. However, in mechanism study of CAD, gene profiles directly bound to ncRNA are rarely explored, especially that ChIRP-RNAseq, a highly sensitive and specific technology to reveal RNA binding RNAs, is also rarely applied. Therefore, in this investigation, we introduced ChIRP-RNAseq to explore the profile of mRNA bound to BTBD7_hsa_circ_0000563.

By means of GO and KEGG enrichment analyses, we found that most enriched cellular component and molecular function of these mRNAs bound to BTBD7_hsa_circ_0000563 were both constituent of ribosome and these mRNAs may mainly involve in translation and necroptosis processes. Moreover, the most enriched biological process with most significant *P* value of these mRNAs was SRP-dependent cotranslational protein targeting to membrane.

The signal recognition particle (SRP), a predominant and universally conserved protein delivery machine, can combine with ribosome and signal peptide, suspend translation and transport the free ribosome – nascent polypeptide chain to the target membrane, which is defined as the cotranslational translocation process [38]. Correct protein function depends on proper localization of proteins, and it is the cotranslational translocation process that is able to ensure that the ongoing protein translation complex can be deliver to the correct cellular or subcellular compartment [39]. Protein is the basis for maintaining normal life activities. It is accepted that the disorder of protein translation will lead to a series of cardiovascular diseases such as cardiac hypertrophy and failure, ischemic heart diseases, atherosclerosis, and hypertension [40, 41]. Therefore, BTBD7_hsa_circ_0000563 is likely to affect the protein translation by regulating the SRP-dependent cotranslation process, and ultimately interfere with the occurrence and development of CAD. In addition, the necroptosis pathway was significant enriched in our results as well. Necroptosis is a form of programmed necrotic cell death, having a significant impact on diseases, of which receptor-interacting serine-threonine kinase 3 (RIPK3) and mixed lineage kinase domain-like (MLKL) act as key mediators [42]. Karunakaran et al. [43] have confirmed that low-density lipoprotein can bring about atherosclerosis through increase RIPK3 and MLKL transcription and phosphorylation, which are two critical steps in the execution of necroptosis [42]. On this basis, they find that the necroptosis inhibitor Nec-1 can reduce atherosclerotic lesion size and markers of plaque instability, including necrotic core formation. Furthermore, Hu et al. [44] find that plasma RIPK3 levels of patients with CAD were significantly higher than those of controls and plasma RIPK3 levels increased linearly with the severity of CAD. According to the evidence above, BTBD7_hsa_circ_0000563 might be able to influence the initiation and progression of CAD via regulating the necroptosis pathway.

After constructing the interaction network of mRNAs bound to BTBD7_hsa_circ_0000563, interaction clusters and hub genes were identified. Although the integral interaction network can reflect the overall connection, the subdivided clusters can present more details of mRNAs bound to BTBD7_hsa_circ_0000563. For example, the functional enrichment analysis of clusters showed that, in addition to translation and necroptosis, other processes that mRNA bound to BTBD7_hsa_circ_0000563 might take part in, such as post-translational protein modification [45, 46], glucose homeostasis [47, 48], neutrophil and lymphocyte [49, 50], and FoxO signaling pathway [51], can make an impact on CAD or atherosclerosis as well.

RPS3 (Ribosomal Protein S3) and RPSA (Ribosomal Protein SA), hub genes of mRNAs bound to BTBD7_hsa_circ_0000563, are both genes encoding ribosomal proteins [52, 53]. In addition to being involved in translation as a component of ribosomes, they also have an undeniable impact on DNA repair and apoptosis [54–56], which are important pathogenesis of atherosclerosis [57, 58]. Furthermore, researchers reported that RPS3 is an essential subunit of nuclear factor-kappaB (NF-κB) and influences rapid cellular activation responses or target genes transcription of NF-κB [59, 60]. Pertschy et al. found that RPS3 can further interact with the NF-κB inhibitor IκBα, regulating the activity of the NF-κB signaling pathway [61]. Interestingly, mounting researchers have confirmed that regulating NF-κB signaling pathway, an important signaling pathway involved in immune and inflammatory reactions, can reduce atherosclerosis and protect coronary artery [62–64]. This makes RPS3 of great research value in CAD. In addition, another researcher believed that RPS3 can enhance cell stress and lead to cell apoptosis by activating c-Jun N-terminal kinase (JNK) [65]. The JNK signaling pathway had also been confirmed to be involved in the foam cell formation in atherogenesis [66]. With regard to RPSA, the protein encoded by RPSA is also known as 37-kDa laminin receptor precursor/67-kDa laminin receptor [53], playing an important role in cell adhesion and signaling transduction [67, 68]. Li et al. corroborated that RPSA can act as a cell surface receptor, binding to pigment epithelium-derived factor (PEDF) to inhibits angiogenesis by inducing endothelial cells apoptosis and reducing cell migration [69]. Of note, PEDF is not only proved to prevent atherosclerosis by inhibiting thrombosis or inhibiting inflammatory reaction of vascular endothelial cells caused by oxidative stress in vivo and in vitro experiments [70, 71], but also proved to be significantly overexpressed in CAD patients in the population, which may act as a protective response and biomarker of CAD [72–74]. Recently, small molecule inhibitors have gradually become a new hot topic in the treatment strategy of chronic diseases. Polyphenol (−)-epigallocatechin-3-O-gallate (EGCG), one such small molecule, has been reported to suppress lipopolysaccharide-mediated inflammation in human vascular endothelial cells by binding to cell surface receptor RPSA [75, 76]. This reveals the vascular protective value of RPSA.

As predicted target miRNA of PRS3, hsa-miR-493-5p has been proven to be closely related to the pathological mechanisms of various cancers [77, 78] and osteoporosis [79]. Simultaneously, hsa-miR-493-5p shows its ability to combine with circRNA and lncRNA to influence diseases development [79–81]. Although hsa-miR-493-5p has not been fully investigated in CAD, its research prospects are limitless.

To sum up, the above evidence implies that BTBD7_hsa_circ_0000563 together with mRNA bound to it may have an impact on initiation and progression of CAD by participating in translation and necroptosis mainly. The present study might provide innovative ideas for further research on the occurrence and development of CAD.

The present investigation had several limitations. First, the small sample size in our study was not enough to minimize the experimental bias and contingency of the results. The mRNAs directly bound to BTBD7_hsa_circ_0000563 should be continuously verified in a large-scale and multicentre cohort to increase the credibility of the results. Second, in our present study, the exploration of mRNAs bound to BTBD7_hsa_circ_0000563 was still in the qualitative stage. In order to further clarify the application value of these mRNAs in the real world, it is necessary to verify the potential expression of these mRNAs, especially the hub genes, in the patients and healthy populations in the future. Third, the mechanisms in which these mRNAs involved were only based on the bioinformatic analysis. Therefore, for ultimately benefiting clinical transformation, rigorous in vivo and in vitro experiments of mRNAs bound to BTBD7_hsa_circ_0000563 should be formulated and implemented to reveal their deeper mechanism and function in CAD.

## Conclusions

In conclusion, a total of 221 mRNAs directly bound to BTBD7_hsa_circ_0000563 were identified in PBMCs of CAD patients in the present investigation through ChIRP-RNAseq. The functional enrichment analysis suggested that these mRNAs might mainly located in ribosome and take part in translation and necroptosis pathways. Based on the interaction network of these mRNAs, RPS3 and RPSA were presumed as hub genes and eight subclusters were analysed. And hsa-miR-493-5p was considered to be the target miRNA of RPS3. These findings may shed light on the possible potential mechanism of BTBD7_hsa_circ_0000563 influencing CAD, and provide innovative research ideas for the mechanism and diagnosis and treatment strategy of CAD.

### Supplementary Information

**Additional file 1.**

## Data Availability

The data discussed in this publication have been deposited in NCBI’s Gene Expression Omnibus and are accessible through GEO Series accession number GSE239635 (https://www.ncbi.nlm.nih.gov/geo/query/acc.cgi?acc=GSE239635). Reviewer access KEY: udodoqaozxwnlyn.

## Abbreviations

CAD: Coronary artery disease
PBMC: Peripheral blood mononuclear cell
GO: Gene ontology
KEGG: Kyoto encyclopedia of genes and genomes
miRNAs: microRNAs
RBPs: RNA binding proteins
ChIRP-RNAseq: Chromatin isolation by RNA purification followed by RNA sequencing
FDR: False discovery rate
DEGs: Differentially expressed genes
PPI: Protein–protein interaction
BP: Biological process
CC: Cellular component
MF: Molecular function
MCC: Maximal clique centrality
EPC: Edge percolated component
MAPK: Mitogen-activated protein kinase
ceRNAs: Competitive endogenous RNAs
ORF: Open Reading frame
IRES: Internal ribosome entry site
RIPK3: Receptor-interacting serine-threonine kinase 3
MLKL: Mixed lineage kinase domain-like
SRP: Signal recognition particle
RPS3: Ribosomal protein S3
RPSA: Ribosomal protein SA
JNK: C-Jun N-terminal kinase
PEDF: Pigment epithelium-derived factor
EGCG: (−)-epigallocatechin-3-O-gallate
