# Supplementary material for: Exploration and bioinformatic prediction for profile of mRNA bound to circular RNA BTBD7_hsa_circ_0000563 in coronary artery disease
Source: BMC Cardiovasc Disord. 2024 Jan 24;24:71. doi: 10.1186/s12872-024-03711-7 (PMC10809658; doi:10.1186/s12872-024-03711-7)
Supplement: Supplementary file 1 — Additional file 1. [file 12872_2024_3711_MOESM1_ESM.docx]

**Supplement to materials and methods**

***PBMCs isolation***

1. Ten ml of artery blood samples were drawn from the arterial sheaths in all subjects by artery puncture before coronary angiography. Blood samples were stored in EDTA anticoagulation tubes at 4 ℃ and were treated within 24 hours.

2. Blood samples were centrifuged at 4 ℃ and 2000r/min for 10 minutes.

3. Then, for each blood sample, five ml of Lymphocyte Separation Medium (TBD, Tianjin, China) was added to one 15ml centrifuge tube for later use in step 4.

4. Discard the supernatant. An equal volume of Phosphate Buffered Saline (PBS) was added to the sediment, then gently blow and resuspend the sediment using a pipette. Slowly drip the resuspended and diluted blood sample along the centrifuge tube wall onto the surface of the lymphocyte separation medium prepared in step 3, making the blood sample float on the surface of the lymphocyte separation medium.

5. Each sample treated in step 4 was centrifuged at 4 ℃ and 2000r/min for 20 minutes. The up speed of centrifuge was set to 4 and the down speed was set to 3.

6. Suck the substance like white film between the upper and middle layers into a new 15ml centrifuge tube carefully.

7. Add PBS to the substance sucked in step 6, making the total volume 10ml. Then invert and mix the mixture.

8. Each mixture was centrifuged at 4 ℃ and 2000r/min for 10 minutes.

9. Discard the supernatant. And there should be a cell aggregate at the bottom of the centrifuge tube.

10. Resuspend the cell aggregate using 2ml of Red Cell Lysis Solution (Biosharp, Anhui, China), then incubate in the dark for 15 minutes.

11. Add PBS to the cell suspension after lysis of red cells in step 10, making the total volume 15ml. Then invert and mix the mixture.

12. Each mixture was centrifuged at 4 ℃ and 2000r/min for 10 minutes.

13. Discard the supernatant. And there should be a cell aggregate at the bottom of the centrifuge tube.

14. Resuspend the cell aggregate using 1ml PBS, then transfer the cell suspension to a 1.5ml EP tube.

15. Each cell suspension was centrifuged at 4 ℃ and 12000g for 1 minute.

16. Discard the supernatant. Suck up any residual liquid carefully.

17. Store the cell aggregates at -80 ℃.
